# Rapid alterations of cell cycle control proteins in human T lymphocytes in microgravity

**DOI:** 10.1186/1478-811X-10-1

**Published:** 2012-01-24

**Authors:** Cora S Thiel, Katrin Paulsen, Gesine Bradacs, Karolin Lust, Svantje Tauber, Claudia Dumrese, Andre Hilliger, Kathrin Schoppmann, Josefine Biskup, Nadine Gölz, Chen Sang, Urs Ziegler, Karl-Heinrich Grote, Frauke Zipp, Fengyuan Zhuang, Frank Engelmann, Ruth Hemmersbach, Augusto Cogoli, Oliver Ullrich

**Affiliations:** 1 Institute of Anatomy, Faculty of Medicine, University of Zurich, Winterthurerstrasse 190, 8057 Zurich, Switzerland; 2 Institute of Medical Physics and Biophysics, University of Muenster, Heisenbergstrasse 11, 48149 Muenster, Germany; 3 Department of Machine Design, Engineering Design and Product Development, Institute of Mechanical Engineering, Otto-von-Guericke-University Magdeburg, Universitaetsplatz 2, 39106 Magdeburg, Germany; 4 Institute of Molecular and Clinical Immunology, Otto-von-Guericke-University Magdeburg, Leipziger Str. 44, 39120 Magdeburg, Germany; 5 Center for Microsocopy and Image Analysis, University of Zurich, Winterthurerstrasse 190, 8057 Zurich, Switzerland; 6 KEK GmbH, Kemberger Str. 5, 06905 Bad Schmiedeberg, Germany; 7 School of Biological Science and Medical Engineering, Beihang University, 37 Xueyuan Rd., Beijing 100191, China; 8 Clinic for Neurology, University Medical Center of Johannes Gutenberg-University Mainz, Langenbeckstraße 1, 55131 Mainz, Germany; 9 University of Applied Science Jena, Carl-Zeiss-Promenade 2, 07745 Jena, Germany; 10 German Aerospace Center (DLR), Institute of Aerospace Medicine, Linder Hoehe, 51147, Cologne, Germany; 11 Zero-G Life Tec, Riedhofstrasse 273, 8049 Zurich, Switzerland; 12 Zurich Center for Integrative Human Physiology (ZIHP), University of Zurich, Switzerland

**Keywords:** Adaptive immunity, spaceflight, signal transduction, gravisensitivity

## Abstract

In our study we aimed to identify rapidly reacting gravity-responsive mechanisms in mammalian cells in order to understand if and how altered gravity is translated into a cellular response. In a combination of experiments using "functional weightlessness" provided by 2D-clinostats and real microgravity provided by several parabolic flight campaigns and compared to in-flight-1g-controls, we identified rapid gravity-responsive reactions inside the cell cycle regulatory machinery of human T lymphocytes. In response to 2D clinorotation, we detected an enhanced expression of p21 ^Waf1/Cip1 ^protein within minutes, less cdc25C protein expression and enhanced Ser147-phosphorylation of cyclinB1 after CD3/CD28 stimulation. Additionally, during 2D clinorotation, Tyr-15-phosphorylation occurred later and was shorter than in the 1 g controls. In CD3/CD28-stimulated primary human T cells, mRNA expression of the cell cycle arrest protein p21 increased 4.1-fold after 20s real microgravity in primary CD4^+ ^T cells and 2.9-fold in Jurkat T cells, compared to 1 g in-flight controls after CD3/CD28 stimulation. The histone acetyltransferase (HAT) inhibitor curcumin was able to abrogate microgravity-induced p21 mRNA expression, whereas expression was enhanced by a histone deacetylase (HDAC) inhibitor. Therefore, we suppose that cell cycle progression in human T lymphocytes requires Earth gravity and that the disturbed expression of cell cycle regulatory proteins could contribute to the breakdown of the human immune system in space.

## Introduction

Gravity has been a constant force throughout evolutionary history on Earth. Thus, it is one of the fundamental biological questions, if and how life on Earth requires and responds to gravity at the functional cellular and molecular level. In unicellular organisms, such as *Paramecium *and *Loxodes*, gravity can be perceived rapidly by gravireceptors, which are gravi-sensitive ion channels in the cell membrane or statocyst-like organelles [1]. In mammalian cells, rapid gravi-responsive elements are unknown.

The sensitivity of human cells exposed to reduced gravity has already been suspected for cells of the immune system since the first Apollo missions, where more than half of the astronauts suffered from bacterial or viral infections [2]. In one instance, an astronaut was infected with an opportunistic pathogen, *Pseudomonas aeruginosa*, which rarely causes disease in people with functional immune systems. In crew members of Skylab and Soyuz, a reduced reactivity of blood lymphoid cells has also been observed [3,4]. Recent studies found a subclinical re-activation of the varicella zoster virus (VZV) in astronauts [5,6]. This virus becomes latent in the nervous system after primary infection, but is frequently reactivated in immune suppressed individuals, such as after organ transplantation, or patients suffering from cancer or AIDS. Because of the obvious and severe effects on the human immune system, serious concerns arose whether spaceflight-associated immune system weakening ultimately precludes the expansion of human presence beyond Earth's orbit [7].

In an extension of this fundamental question, it is important to ask if the molecular and cellular structure of human life on Earth may require gravity for regular function and survival, and if therefore gravity-dependent mechanisms will keep us dependent on the gravity field of Earth. Indeed, about one decade later, a pioneering discovery from Cogoli et al. at the first Spacelab-Missions in the year 1983, where isolated human lymphocytes failed to proliferate after several days in microgravity, provided the first strong evidence of cell sensitivity to long-term reduced gravity exposure [8]. Follow-up experiments clearly verified the depression of lymphocyte proliferation activation after mitogenic stimulation in long-term microgravity [9].

During the last two decades, many studies evidenced alterations in molecular mechanisms and signal transduction processes in cells of the immune system as a direct result of reduced gravity [10-30]. For instance, in lymphocytes, microgravity affected protein kinase C [10,11], influenced NF-kB and MAPK-signaling [13], altered the expression of c-fos, c-myc and c-jun (summarized in [14]), reduced the expression of IL-2 receptor [21,22] and decreased the capacity for the production of cytokines [23]. However, the underlying molecular mechanisms are completely unknown. Given the extremely complex nature of cellular signal transduction networks in spatio-temporal dimensions, any observed effect could be secondary, adaptive, driven by negative or positive feedback-loops and thus far beyond the initial and primary cellular response to altered gravity. In order to perform the first step in elucidating the cellular response to microgravity systematically, we aimed to investigate if mammalian cells are rapidly responding to reduced gravity and to discover the most initial and earliest molecular reactions.

The only opportunity to perform experiments with living mammalian cells in reduced gravity without leaving our planet is onboard an aircraft performing parabolic flight manoeuvres which is weightless when it is flying on a Keplarian trajectory, described as an unpropelled body in ideally frictionless space subjected to a centrally symmetric gravitational field. In contrast to the logistic limitations of the International Space Station ISS and other space-based research platforms, parabolic flights provide frequent and repeated access to microgravity and therefore allow replication and modification of experiments within a reasonable time frame, which are not only characteristics, but rather requirements, of modern biomedical research. In the search for rapid-responsive molecular alterations in mammalian cells, short-term-microgravity of 22s provided by parabolic flight manoeuvres on board of the Airbus A300 is an ideal instrument to elucidate these initial and primary effects, widely without the influence or interference of secondary signal cascades or feedback loops.

Because rapid reactions to reduced gravity in mammalian cells have not been investigated so far, a technical equipment and experimental platform to perform such experiments in real microgravity was lacking. For this reason, we recently developed an experimental system which for the first time allows for large-scale cell culture experiments with living mammalian cells on board of the parabolic flight aircraft Airbus A300 ZERO-G [31]. Due to the fact that cells of the immune system are obviously influenced by altered gravity [8-30], its gravisensitive nature renders these cells an ideal biological model in the search for primary gravisensitivity in mammalian cells.

Although access to microgravity provided by parabolic flights is faster, more frequent and less complicated than access to the microgravity of space, parabolic flights are far from being a readily available day-to-day method of gravitational research. Thus, scientists have been innovative in order to find experimental approaches to study the influence of gravity without access to space or to special flight manoeuvres. For this reason, so-called 2D clinostats complement the gravitational research platforms by a solely-ground-based device, which enable the rotation of a sample around one axis perpendicular to the gravitational field [32]. The condition of weightlessness is characterized by the lack of sedimentation and thus by a homogeneous distribution of particles. On ground, this situation can be achieved by rotating a suspension of particles, which will still fall, but will be also forced on circular paths with decreasing radii through faster rotation of the system. The clinostat rotation has to be fast enough to achieve a situation where the rotated system no longer perceives the rapidly turning gravity vector (compensation of the gravitational force) and thus experiences "weightlessness" [33-37].

In a combination of experiments using "functional weightlessness" provided by 2D clinorotation and real microgravity provided by several parabolic flight campaigns, we investigated rapid graviresponsiveness at the molecular level in human T lymphocytes, the key player of the specific immune response. Our aim was to identify a rapidly reacting gravity-responsive element in mammalian cells.

## Materials and Methods

### General experimental procedure

1.) Functional weightlessness: The ground-based studies in "functional weightlessness" were reviewed by the European Space Agency ESA (ESA-CORA-GBF-2005-005) and performed under an ESA contract in collaboration with the German Aerospace Center (DLR) using a 2D test tube clinostat. Due to the present technical limitation of the used DLR clinostat device, ultra-short clinorotation experiments (less then 1 min incubation time) were not possible. Therefore, we focused on a time frame which allows comparison of experiment results with a follow-up study on board of a sounding rocket, providing 5 min microgravity and which is scheduled for the upcoming MASER-12 mission in February 2012 (based on ESA-AO-2004-010) 2.) Real microgravity: In search of rapid-responsive molecular alterations in mammalian cells, short-term-microgravity provided by parabolic flight manoeuvres is an ideal instrument to elucidate initial and primary effects, without the influence and interference of secondary signal cascades. Parabolic flights provide 1 g, microgravity and hypergravity conditions. The influence of hypergravity (1.8 g) was tested separately on a labortatory centrifuge (MUSIC, multi-sample centrifuge). For parabolic flight experiments and especially for this study, we developed and constructed a complete experimental system which allows cell culture experiments with living mammalian cells in microgravity on board the Airbus A300 ZERO-G aircraft. Experiments in real microgravity were reviewed by DLR and ESA and performed during five parabolic flight campaigns provided by DLR and ESA. The parabolic flight experiments were reproduced during different independent flights and different independent flight campaigns. Analysis of μg samples and 1 g in-flight-controls were performed using qRT-PCR.

### Experiments in "functional weightlessness": Cell culture, stimulation and sample preparation

Human Jurkat T-cells (ATCC TIB-152) were cultured in RPMI-1640-medium (Sigma-Aldrich, Germany), supplemented with 10% FCS (Biochrom, Germany) and penicillin/streptomycin (Sigma-Aldrich, Germany). Stimulation was performed using 10 ng/ml soluble CD3 (BD Bioscience, Heidelberg, Germany) and 0.5 μg/ml soluble CD28 antibodies (BD Bioscience, Heidelberg, Germany) or alternatively by 10 ng/ml phorbolmyristylacetate (PMA, Sigma-Aldrich, Germany) at a cell density of 10^6 ^cells/ml under the conditions of clinorotation. 1g-control experiments have been performed inside the clinostat, but without rotation. Cell suspensions were carefully mixed with the activator solution (CD3/CD28 or PMA) or control solution (only medium) and filled in the incubation tubes (sterile and sealed 1 ml pipettes, Falcon) by an automatic pipette in order to avoid cell shearing or damage. The time interval needed to stimulate cells prior to the start of altered gravity (mixing and filling the incubation chamber) as well as the time needed to harvest cells following altered gravity (removal of the seal and transfer into ice-cold PBS) was kept as short as possible and constant over all samples. Under the chosen experimental conditions (60 rpm, pipette diameter 4 mm), a maximal residual acceleration of 4 × 10^-3 ^g is achieved at the border of the pipette, which decreases towards the center. The clinostat was placed within an incubator thus providing constant temperature conditions of 37°C during the experiments. After clinorotation, the reaction was stopped immediately by the addition of ice-cold PBS (Sigma-Aldrich, Germany). For preparation of total cell lysates, cells were harvested in ice-cold PBS (Sigma-Aldrich, Germany), centrifuged (300 g for 10 min), washed twice in ice-cold PBS and stored as dry pellets at -80°C. At least 3 independent clinorotation experiments were performed.

### Sample analysis

For analysis of phosphorylation and expression of signal molecules after clinorotation, cell lysates were analysed by phospho-specific antibodies in immunoblots and mRNA expression by quantitative RT-PCR. All antibodies were from Cell Signaling Technology, Danvers, MA. Quantitation was performed by Gene Profiler (Version 3.56, Scananlytics Inc.) or Image J software (National Institutes of Health, USA). Data were analysed by one way ANOVA, followed by the Bonferroni test for comparison of certain column pairs. p < 0.1 was considered to be significant, p < 0.01 as very significant and p < 0.001 as extremely significant.

### RNA isolation and cDNA synthesis for clinorotation samples

After clinorotation of cells for 5, 10, and 15 min, Trizol (life technologies, Darmstadt, Germany) was added to stop the reaction and lyse the cells and RNA was isolated according to the manufacturer's protocol. RNA was subsequently purified using the RNeasy Mini kit (Qiagen, Hilden, Germany) including the recommended DNase digestion. RNA concentration and purity (260/280 ratio) was measured in a Nanodrop spectrophotometer (Thermo Scientific, Germany). 200 ng of RNA were reverse transcribed using the SuperScript First-Strand Synthesis System (Invitrogen, Karlsruhe, Germany) and cDNA was used for TaqMan Gene Expression assays specific for p21 and beta-2-microglobulin (Applied Biosystems/life technologies, Darmstadt, Germany). Real time PCR analysis was performed on a light cycler 480 (Roche Applied Science, Mannheim, Germany).

### Parabolic Flight experiments

The scientific community in Europe shares only one larger aircraft which is licensed for parabolic manoeuvres, an Airbus A300 built in 1973, which is operated out of the Bordeaux-Mérignac airport (France) or from Cologne airport (Germany) by the company Novespace and is flying up to 9 campaigns each year [31]. Experiments have been performed during the 8th, 9th,10th and 13th DLR Parabolic Flight Campaign and during the 45th ESA Parabolic Flight Campaign inside the Airbus A300 ZERO-G, a specially-configured test aircraft (NOVESPACE, Bordeaux, France), and under the standing orders of NOVESPACE (A300 ZERO-G Rules and Guidelines RG-2001-1, RG-2008-1, RG-2008-2, RG-2009-1 and RG-2009-02) and the CEV (Centre d'essai en vol).

### Development of in-flight-hardware

**Figure 1:**
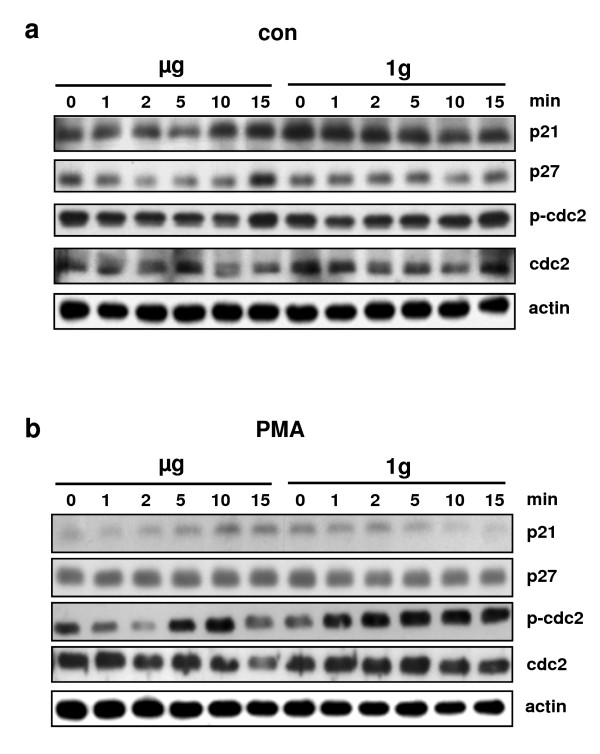
**Protein expression of p21 and p27 and phosphorylation and protein expression of cdc2 in Jurkat T cells after 1-15 min simulated weightlessness (μg) without (con, **a**) and with stimulation with PMA at the onset of simulated weightlessness (PMA, **b**)**. 1g-control experiments have been performed inside the clinostat, but without rotation. 5 independent experiments were performed and pooled proteins were analyzed. One representative beta-actin control is shown.

For the in-flight experiments on board the Airbus A300 ZERO-G, we developed an experimental system, which allows cell culture tests with living mammalian cells in microgravity. During the detailed design of the main and secondary functional elements the basic design, rules "simple" (e.g. use of a module system for the rack design, only 15% of the required components are specially made), "clear" (e.g. principle of the liquid flow did not lead to indefinable conditions) and "safe" (e.g. redundant arrangement of parts absorbing forces, double sealing of liquids) were met. Primary importance was placed on realising the direct safety technique during the development activity. The experimental structure consists of three experiment racks (*storage rack *for cell culture containers before the experiments, *cooling rack *for storage of cell culture containers after the experiments and a *working rack *for execution of the experiments, see Figure 1). All modules have been developed for performing experiments with living mammalian cells during parabolic flights and allow storage of cell culture compartments until the start of the experiment, injection of a fluid (activator) at the beginning of each parabola and automatic injection of a second fluid (fixation) after 20s at the end of each parabola. Appropriate in-flight controls have been obtained during the 1 g flight phase directly before or after the parabola. The module system can accommodate 60 cell culture containers for each flight. Injection of all fluids operates automatically and is pre-programmed, while exchange of cell culture containers and supervision of the experiment is done by the researchers during the parabolic flight. For a technical description of the in-flight-hardware, please refer to the additional file 1 (short technical description of in-flight-hardware).

### Cell culture and preparation for parabolic flights

Human Jurkat T-cells (ATCC TIB-152) were cultured in RPMI-1640-medium (Sigma-Aldrich, Germany), supplemented with 10% FCS (Biochrom, Germany) and antibiotics. PBMCs were isolated from the buffy coat fraction from voluntary healthy donors (blood donor service Zurich, Switzerland) using Ficoll Hypaque density gradient centrifugation. CD4^+ ^T-lymphocytes were isolated from PBMCs using a commercially available MACS-separation system (IMAG, BD Bioscience). For each experiment, a separate blood donor was used. In-flight μg and 1 g control experiments have been performed in special in-flight cell culture bags (Nutrimix, Braun, Melsungen, Germany) containing 3 × 10^7 ^cells in 15 ml medium per bag. In some flight experiments, 50 μM of the histone acetyltransferase (HAT) inhibitor curcumin [38], 100 μM of the histone deacetylase (HDAC) inhibitor valproic acid or 10 μM of the poly-ADP-ribose-polymerase-1 (PARP-1) inhibitor 5-aminoisoquinoline (AIQ) were added.

### Cell and sample transports

Transport of cell culture bags with living cells in in-flight-configuration and of fixed samples after the parabolic flight was carried out by military transport flights (Beech 1900D, Swiss Air Force) from Zurich to Bordeaux at the appropriate temperature during each flight day of the 13^th ^DLR Parabolic Flight Campaign. During the 9^th ^DLR and 45^th ^ESA Parabolic Flight Campaign in Bordeaux, they were transported to Bordeaux by commercial flights inside the passenger cabin with special permission. During the 10^th ^DLR Parabolic Flight Campaign in Cologne, Germany, cell cultivation and preparation could be performed on-site at the Biomedical Science Support Center (BC) of the German Aerospace Center (DLR). After arrival at the flight location the evening before the flight, cells were incubated overnight at 37°C and all handled very carefully in order to avoid any mechanical or temperature cell stress before the flight. All steps of the entire cell preparation and transport procedure have been tested extensively in respect to cell viability and function beforehand (data not shown). All procedures during the Parabolic Flight Campaigns have been tested several times, are highly standardized and follow an extensive and detailed standard protocol. During the campaign, all procedures have been documented and double-checked.

### Experimental procedures

In-flight μg and 1 g control experiments were performed in special in-flight cell culture bags containing 3 × 10^7 ^cells in 15 ml medium per bag. During the onset of μg or during 1 g (in-flight control experiments), 10 ng/ml PMA or 10 ng/ml CD3 and 0.5 μg/ml CD28 or cell culture medium (non-stimulated cells) were added. After 20 s of μg or 1 g, cells were fixed by addition of 70% ethanol (final concentration) and immediately cooled (4°C) during the remaining flight. Experiments were performed at least 3 times during independent flights and separate flight days. After the flight, cells were transported to the laboratories, harvested and subjected to analysis.

### Parabolic flight manoeuvres

During a flight campaign, which normally consists of three individual flights, 31 parabolas are flown on each flight, with 93 parabolas in total. On each parabola, there is a period of increased gravity (1.8 g) which lasts for 20 seconds immediately prior to and following the 20 seconds period of reduced gravity (acceleration in x-, y- and z-axis was below 2 × 10^-3^g at all times during the 0 g parabola). During the Parabolic Flight manoeuvre, the aircraft gradually pulls up its nose and starts climbing at an angle of approximately 45 degrees. This phase lasts for about 20 seconds, during which the aircraft experiences an acceleration of around 1.8 g. The engine thrust is then reduced to the minimum required to compensate for air-drag, and the aircraft is then in a free-fall condition, lasting approximately 20 seconds, during which weightlessness is achieved. At the end of this phase, the aircraft must pull out of the parabolic arc, a manoeuvre which gives rise to another 20-seconds period of 1.8 g on the aircraft, after which it returns to normal level flight attitude. Special designated flight areas were above the Atlantic Ocean and the Mediterranean Sea. Three researchers executed the experiments on board during each flight. Two loaded and unloaded the cell containers in the working rack within 60s of the 1 g phase between each parabola. A third researcher was in charge of operating the control unit and monitoring the subsystems. Each was trained to overtake any other experimenter's position in a case of emergency. All researchers on board were medically approved for parabolic flights by the Parabolic Flight Medical Commission, Caen, France.

### RNA isolation and cDNA synthesis

360 μl of Trizol were added to each frozen cell pellet and homogenized. After incubation for 5 min at room temperature, the cell suspension was centrifuged for 10 min at 17000 rpm and 4°C. 5 μg of linear acrylamide (Ambion/Applied Biosystems, Darmstadt, Germany) was added to the supernatant and vortexed for 10 sec. 72 μl of chloroform were added and the suspension was vortexed again before centrifugation at 17000 rpm for 5 min. The aqueous phase was mixed with 0.8 vol isopropanol and RNA was precipitated for 90 min at -20°C. After precipitation of the RNA by centrifugation for 30 min at 17000 rpm and 4°C, the supernatant was removed and the pellet was washed with 70% ethanol, air dried, and re-dissolved in H_2_O. A DNaseI digestion was performed (RNase free DNase set; Qiagen, Hilden, Germany) according to manufacturer's instruction. RNA was purified by phenol-chloroform extraction and the RNA was subsequently precipitated by addition of 1/10 vol 3 M Na-acetate (pH 4.8), 1 vol isopropanol and incubation for 15 min at -80°C. The RNA was pelleted by centrifugation at 17000 rpm for 15 min at 4°C and washed twice with 70% ethanol. The RNA pellet was resuspended in H_2_O and the concentration was measured photometrically. 1 μg of RNA was reverse transcribed using the RevertAid H Minus First Strand cDNA Synthesis Kit (Fermentas, St. Leon-Rot, Germany) and random hexamer primer according to the manufacturer's protocol.

### Measurement of RNA quality

RNA quantity and purity was measured with a spectrophotometer. Depending on the cell type, concentrations varied between 10 and 544 ng/μl. RNA yields from primary T cells were usually low compared to RNA yields from Jurkat T cells. RNA purity (260/280 ratio) ranged between 1.7 and 2.0, sufficient for further analyses like reverse transcription and real time PCR. Exemplary samples were analyzed for the RNA integrity on a 2100 Bioanalyzer. RNA integrity numbers (RIN) between 2.4 and 3.2 were measured for the analyzed samples. We added this informations in the material and methods section.

### Quantification by real time PCR and statistical analysis

1 μl of cDNA was used per real time PCR sample and was added together with the primer for the gene of interest and the reference gene respectively to the SYBR Green PCR Master Mix reaction (Applied Biosystems Darmstadt, Germany). Real time PCR was performed on the 7500 Real Time PCR System (Applied Biosystems, Darmstadt, Germany). The PCR primers used for the study included: 5'-TGAGTGCTGTCTCCATGTTTGATG-3' and 5'-TGCTCCCCACCTCTAAGTTGC-3' specific for β2 microglobulin as a reference gene and 5'- TGGAGACTCTCAGGGTCGAAAA-3'and 5'-GGATTAGGGCTTCCTCTTGGAGA-3'for the gene of interest p21. For each cDNA sample triplet reactions were performed with p21 and β2 microglobulin specific primers and differences in gene expression levels were calculated according to the Pfaffl method [39]. Subsequently, the Wilcoxon signed rank test was applied to analyze the significance of the detected difference in expression levels.

## Results

### Experiments in simulated weightlessness (2D clinorotation)

**Figure 2:**
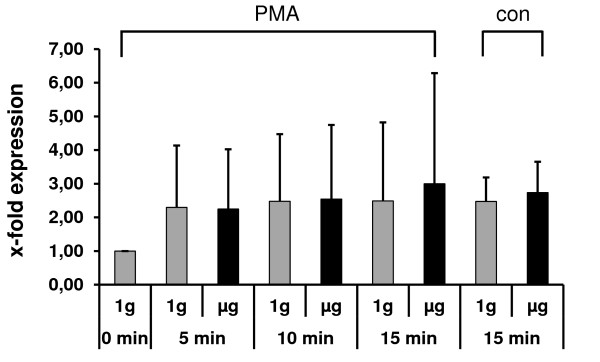
**mRNA expression of p21 ^Waf1/Cip1 ^in Jurkat T cells after 5, 10, and 15 min of simulated weightlessness (μg) by clinorotation**. PMA was added at the onset of weightlessness for cellular stimulation. 1 g control experiments were performed in the clinostat without rotation. Control experiments (con) were performed for the time point 15 min with medium substituting the PMA. 3 independent experiments were performed.

**Figure 3:**
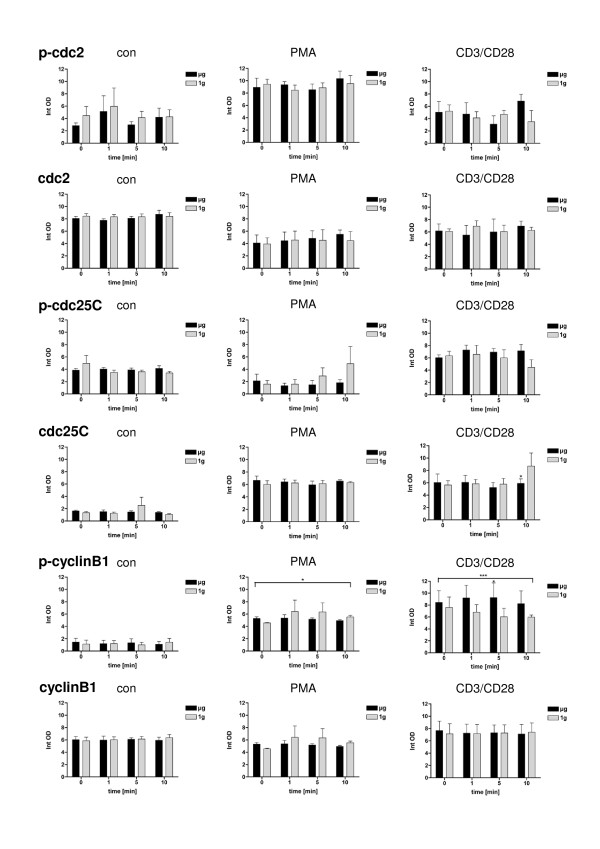
**Protein expression and phosphorylation of cdc2, cdc25 and cyclinB1 in Jurkat T cells at the onset (0 min) and after 1 min, 5 min and 10 min simulated weightlessness (μg) without **(con) **or with PMA (PMA) or CD3/CD28 soluble antibodies (CD3/CD28)**. 1g-control experiments have been performed inside the clinostat, but without rotation. Three independent experiments were performed and analysed separately by immunoblotting. Quantitation was performed by Gene Profiler software. The results are expressed as mean ± SEM. Data were analysed by one way ANOVA, followed by Bonferroni test for comparison of certain column pairs. p < 0.1 = *, p < 0.01 = **, p < 0.001 = ***

Because long-term *in vitro *studies clearly revealed that T cells lost their proliferative capacity in microgravity [8,9], we first investigated key molecules of cell cycle control in short-term simulated weightlessness provided by 2D clinorotation of PMA-activated or non-activated human Jurkat T lymphocytes (Figure 1, 2, 3). The first set of experiments aimed to give a first impression on possible rapid and early alterations in the cell cycle control machinery in T cells. Due to the construction principle of the DLR-clinostat, reliable incubation times are minutes long and therefore, technically we were not able to perform clinorotation experiments with shorter incubation times than 1 min.

### Protein expression of p21 and p27 and phosphorylation and protein expression of cdc2 in Jurkat T cells in simulated weightlessness

In a first set of experiments, we investigated the protein expression of the cell cycle inhibitory proteins p21 ^Waf1/Cip1 ^and p27 ^Kip1 ^(Figure 1). We further investigated Tyr15-phosphorylation of cdc2 (Figure 1), which is a critical regulatory step in activating cdc2 during cell cycle progression into mitosis [40]. After treatment of Jurkat T cells with PMA, p21 ^Waf1/Cip1 ^protein expression was reduced 4.2-fold after 15 min in 1 g, but was enhanced 1.6-fold after 15 min during clinorotation. p21 ^Waf1/Cip1^. Protein expression during clinorotation was 4.6-fold higher compared to 1 g controls after 15 min incubation with PMA. p27 ^Kip1 ^protein expression during clinorotation was 1.4-fold higher compared to 1 g controls after 15 min incubation without PMA stimulation. Tyr15-phophorylation of cdc2 increased after incubation with PMA in clinorotated as well as in 1 g control samples. However, during clinorotation Tyr-15-phosphorylation occurred later and was shorter than in the 1 g controls. As a result of these clinostat experiments, p21 ^Waf1/Cip1 ^protein expression is possibly dependent on gravity conditions and therefore represents a candidate for early gravi-sensitive alterations in T cells.

In addition to the protein expression levels (Figure 1), mRNA transcription levels were analyzed for p21 ^Waf1/Cip1 ^in Jurkat T cells by real time PCR using 2D clinorotation for simulating 5, 10, and 15 min of weightlessness (Figure 2). For all time points a 2-3 fold increase in p21 ^Waf1/Cip1 ^expression was observed in 1 g and simulated μg conditions under the influence of PMA. Even in control experiments (con) where PMA was substituted by medium the p21 ^Waf1/Cip1 ^expression level increased 2.4 - 2.7-fold. No difference between the μg and the 1 g group was detectable.

### Protein expression and phosphorylation of cdc2, cdc25 and cyclinB1 in Jurkat T cells during simulated weightlessness

Since cdc25 protein phosphatase is responsible for dephosphorylating and activating cdc2 [41], we investigated Ser216-phosphorylation of cdc25 (Figure 3). When phosphorylated at Ser216, cdc25C binds to members of the 14-3-3 family of proteins, sequestering cdc25C in the cytoplasm, preventing premature mitosis [42]. Phosphorylation of cyclin B1 is required for cdc25C-dependent dephosphorylation of Tyr15 within cdc2 and subsequent cdc2/cyclin B1 activation [43]. Therefore, we systematically investigated phosphorylation of cdc2 (Tyr15), cdc25 (Ser216) and cyclinB1 (Ser147) in Jurkat T cells after stimulation with PMA or CD3/CD28 antibodies in simulated weightlessness provided by clinorotation compared with 1 g controls (Figure 3). In the next set of experiments, we detected less cdc25C protein expression after 10 min stimulation with CD3/CD28 in the presence of clinorotation compared to 1 g controls. Additionally, on average over all time points, Ser147-phosphorylation of cyclinB1 was reduced during clinorotation after PMA stimulation and enhanced after CD3/CD28 stimulation. Other alterations in comparison of clinorotated and 1 g control samples could not be detected and, despite some slightly significant gravity-dependent effects in cdc25C protein expression after 10 min, there were no substantial differences in phosphorylation of cdc2 (Tyr15), cdc25 (Ser216) and cyclinB1 (Ser147) in Jurkat T cells between clinorotated and 1 g control samples in the time frame of 1-10 min.

In our experiments using "functional weightlessness" provided by a 2D-clinostat, we found that p21 ^Waf1/Cip1 ^protein expression was distinctly higher in clinorotated samples than in 1 g control samples after incubation with PMA. Since detection of p21 ^Waf1/Cip1 ^protein in samples from parabolic flights failed due to the technical and logistical limitations of sample handling and processing during parabolic flight campaigns, we decided to investigate p21 mRNA expression in real microgravity provided during parabolic flights.

### Experiments in real microgravity (parabolic flights)

As alterations of p21 ^Waf1/Cip1 ^and p27 ^Kip1 ^protein expression and of Tyr15-phosphorylation of cdc2 (Figure 1) are probably a rapid response to simulated weightlessness in Jurkat T cells, we further investigated whether these effects could be detected and therefore confirmed in real microgravity provided by parabolic flights. During parabolic flight experiments, cells were activated at the onset of μg (or during 1 g for in-flight control experiments) by addition of PMA or CD3/CD28 (or of cell culture medium for baseline experiments) and fixed 20s after the period of altered gravity.

### Development of the experiment system for the Airbus A300

**Figure 4:**
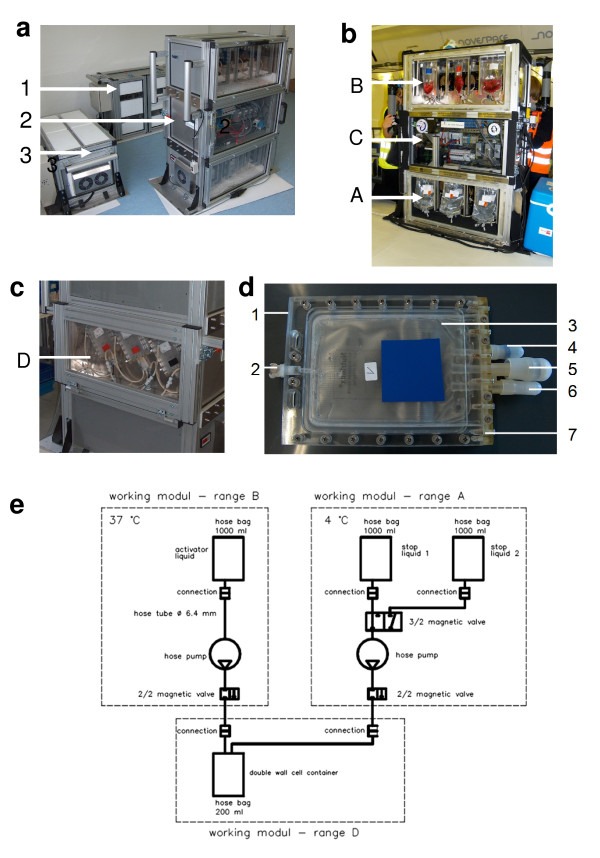
**In-flight-experiment system during parabolic flights on board the Airbus A300 ZERO-G**. **a: **Experiment hardware structure which consists of an incubator rack to store the cell containers temporarily before the experiment at 37°C (1), an experiment rack, in which all active aggregates are accommodated for the execution of the experiment and where the living cells are handled during altered gravity (2) and a cooling rack to store temporarily all cell containers after the injection of the stop/fixation liquid at 4°C until landing (3). **b**. Structure of the working rack, rear side. In range A (4°C) are three separate hose pumps, which pump one of the two possible stop liquids (e.g. ethanol or 4% formaldehyde) into the cell containers. The pumps are activated and regulated by the control unit inside range C, which also carries all electrical connections and fuse elements. All liquids are sucked under exclusion of air. Inside range B (37°C) are three bags with activator liquids, connected to three separate hose pumps, which pump the activator solution into the cell container upon activation of the individual experiment. **c**. Structure of the working rack, front side. Range D is the actual working space with three cell containers in parallel, which are connected to the activator and fixation liquid bag. Range D is also waterproof and the door position is controlled by a safety switch (sensor). Activation of the experiment is only possible when the front door is closed. **d**. Double-wall liquid-proof cell container. A maximum of 60 container can be accommodated during one flight. 1 = plastic container, 2 = air valve, 3 = internal sterile cell culture bag (Nutrimix 0.25l), 4 = connector 1 (activator liquid), 5 = connector 2 (stop/fixation liquid), 6 = connector 3 (filling port for cells, performed preflight), 7 = plastic flange. **e**. Fluid loop diagram for one cell container. The entire experiment system has three identical fluid loops.

The experiment hardware structure is demonstrated in Figure 4 (please refer to the Figure 4 legend and to the additional file 1). It consists of an incubator rack to temporarily store the cell containers before the experiment at 37°C, an experiment rack in which all active aggregates are accommodated for the execution of the experiment and where the living cells are handled during altered gravity and a cooling rack to store temporarily all cell containers after the injection of the stop/fixation liquid at 4°C until landing.

The methodical approach to the development of an experimental system which allows cell culture experiments on board of an Airbus A300 during parabolic flight manoeuvres was performed according to Pahl/Beitz [44] with the four stages: task definition, concept stage, design stage and final solution. The functional engineering description or the overall function to be fulfilled by the system setup was described as follows: A test setup which enables three different cell lines to be mixed, to a large extent homogeneously, with certain activator liquids at the start of the weightlessness phase and a stopping liquid/fixative at end of the weightlessness phase. Appropriate 1 g control experiments had to be executed on board. A main requirement was the fulfilment of all safety requirements. Primarily, it must be ensured that liquids never leak from the test setup during the parabolic flights, which could cause a hazard to the flight personnel as well as damage to the electric systems of the aircraft. Thus, all equipment in contact with liquids must be designed with double liquid-proof walls. A further requirement was the temperature of the cells and activator liquids (37°C) and the temperature of the stop liquid/fixative (4°C). Further points included in the functional engineering description and task definition stage were a.) enable fast and easy equipping with liquids, b.) realisation of the direct safety stage, i.e. leak-proof under the conditions in the aircraft, c.) clear functional sequences, d.) good miscibility of the liquids during the experiment in the cell culture bag, e.) fill under exclusion of air, f.) to a large extent transparent construction for observation whether air inclusions exist, g.) low weight (mass), h.) small space requirement and i.) to fulfil all applicable rules and regulations by the aircraft operator. In the conceptual design stage, the overall function is structured into its sub-functions and their links and efficacy principles were then assigned to the sub-functions. Conventional, intuitive and discursive solution finding methods were used to draw suitable action principles. It was of primary importance that the high safety requirements be fulfilled with all the selected efficacy principles.

### Enhanced p21 ^Waf1/Cip1 ^mRNA expression in real microgravity

**Figure 5:**
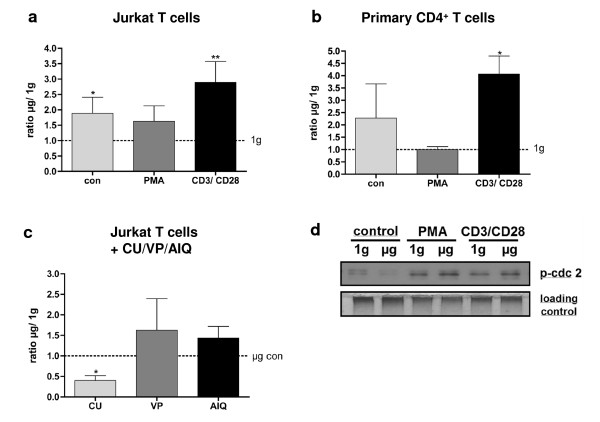
**Expression of p21 ^Waf1/Cip1 ^mRNA in real microgravity provided by parabolic flights in non-stimulated (con) or stimulated (PMA or CD3/CD28) Jurkat T cells or primary CD4^+ ^T cells**. p21 ^Waf1/Cip1 ^mRNA expression is demonstrated as ratio between expression in microgravity (μg) and 1 g in fig.5ab. In fig. 5c, p21 ^Waf1/Cip1 ^mRNA expression is demonstrated as the ratio between expression in microgravity (μg) with a pharmacological inhibitor (VP, CU or AIQ) and without. Three independent experiments were analysed. VP = valproic acid, CU = curcumin, AIQ = 5-aminoisoquinoline. Results are expressed as mean ± SEM. Data were analysed by Wilcoxon test. p < 0.1 = *, p < 0.05 = **. In fig 5d, phosphorylation of cdc2 in Jurkat T cells after 20s real microgravity provided by parabolic flight manouvres in non-stimulated (con) or stimulated (PMA or CD3/CD28) cells is shown. PMA or CD3/CD28 was added at the onset of altered gravity. Loading control: corresponding gel stained by Coomassie after blotting. Five independent experiments during different flight days were performed and pooled protein was analysed.

Jurkat T cells and primary T cells were exposed to 20s of microgravity during parabolic flights and analysed for their differential gene expression of p21 ^Waf1/Cip1 ^(cyclin-dependent kinase inhibitor 1) which functions as a regulator of cell cycle progression at the G_1 _phase by directly inhibiting the activity of cyclinE/CDK2 and cyclinD/CDK4 complexes (Figure 5). Three different conditions were tested: 1.) medium was injected as a control solution to identify effects of microgravity on gene expression without stimulation (con), 2.) PMA was used to activate directly the signal transduction enzyme protein kinase C (PKC) and 3.) CD3/CD28 antibodies were applied to stimulate the cells via their T cell receptor (TCR) and CD28 receptor (CD3/CD28). Comparison of 1 g and μg showed that even for non-stimulated conditions, an increased p21 ^Waf1/Cip1 ^gene expression (1.9 fold in Jurkat T cells and 2.3 in primary CD4^+ ^T cells, Figure 5ab) is detectable. In CD3/CD28-stimulated Jurkat T cells and primary CD4^+ ^T cells, increased p21 ^Waf1/Cip1 ^gene expression was upregulated 2.9-fold and 4.1-fold, respectively (Figure 5ab).

### Histone acetylation-dependent p21 ^Waf1/Cip1 ^mRNA expression in real microgravity

Because histone acetylation is described as one of the regulators of p21 ^Waf1/Cip1 ^gene expression, we investigated the effect of the histone acetyl-transferase (HAT) inhibitor curcumin and the histone deacetylase (HDAC) inhibitor valproic acid on microgravity-triggered p21 ^Waf1/Cip1 ^gene expression. We found that curcumin abrogated the microgravity-induced p21 ^Waf1/Cip1 ^gene expression, whereas valproic acid had the opposite effect (Figure 5c). Additionally, the poly-ADP-ribose-polymerase-1-inhibitor 5-aminoisoquinoline (AIQ) had no significant effect on microgravity-induced p21 ^Waf1/Cip1 ^gene expression (Figure 5c). The use of genetically modified organisms or siRNA knockdown methods was prohibited on board the Airbus A300 ZERO G and therefore not possible (remark: the use of genetically modified organisms is now possible after approval by the French authorities).

### Enhanced Tyr15-phosphorylation of cdc2 in real microgravity

We could detect an enhanced Tyr15-phosphorylation of cdc2 in PMA- and in CD3/CD28 stimulated Jurkat T cells after 20s real microgravity, but not in non-stimulated cells (Figure 5d). In microgravity, Tyr15-phosphorylation of cdc2 after addition of PMA or CD3/CD28 was enhanced 1.44-fold or 1.35-fold, respectively, compared to 1 g-in-flight-controls. Without stimulation, Tyr15-phosphorylation of cdc2 was reduced 1.85-fold in microgravity. Due to the technical and logistical limitations of sample fixation and sample transport after a parabolic flight, we were not able to detect p21 ^Waf1/Cip1 ^or p27 ^Kip1 ^protein in the flown samples by commercially available antibodies.

In conclusion, we detected an enhanced expression of p21 ^Waf1/Cip1 ^protein within minutes of clinorotation and an enhanced expression of p21 ^Waf1/Cip1 ^mRNA within 20s of real microgravity, which could be abrogated by the HDAC inhibitor curcumin. Additonally, we found an enhanced Tyr15-phosphorylation of cdc2 in real microgravity. Therefore, we identified a rapid effect of microgravity on the expression of a cell cycle regulatory gene in mammalian cells.

## Discussion

Because gravity is present everywhere on Earth, scientists and engineers have to be highly innovative in order to find experimental approaches for studying the influence of gravity without leaving our planet. Utilization of microgravity research platforms requires long-term planning, technical innovations and extraordinary safety efforts. Beyond the technical and logistical challenges, results arising from such experiments frequently do not end up in a prompt follow-up experiment, as new proposals are often required, long waiting times are prevalent, and administrative and logistical challenges have to be solved. Therefore, it is difficult to perform biomedical research using microgravity platforms with the same rigorous logical thoroughness as on ground, and gaps in the logical chain of reasoning often cannot be filled due to limitation of research platforms, both in terms of availability and in terms of the technical and logistic framework.

The validity of the 2D fast-rotating clinostat has been shown through a direct comparison of data from simulation experiments with that from space experiments using T-lymphocytes [9,21,45-47]. In general, results from comparative studies obtained so far (space and fast-rotating 2D clinostat experiments) are similar, though the response in real microgravity appears to be more pronounced and/or faster [32]. Experiments on board parabolic flights using the Airbus A300 aircraft are an optimal research platform to investigate initial alterations of cellular mechanisms in microgravity without the influence or interference of secondary signal cascades or feedback loops. For this reason, we developed a new experimental system, which for the first time allows for large-scale cell culture experiments with living mammalian cells onboard the parabolic flight aircraft Airbus A300 ZERO-G. During parabolic flight manoeuvres, a repeated state of "weightlessness" is achieved. However, true weightlessness (= zero gravity) does not exist due to the presence of masses in our universe. Even at an altitude of e.g. 350 km, which corresponds to the orbital position of the ISS, the level of gravity is still 9.04 ms^-2 ^compared to 9.81 ms^-2 ^at the Earth's surface. But if the gravitational and centrifugal forces are equal in amount, but opposite in their vectors, free-fall ("weightlessness") condition is achieved for a spacecraft. This situation is named „microgravity" because of the residual acceleration in the range of 10^-4 ^- 10^-6 ^g. Whereas sedimentation, hydrostatic pressure and convection are linearly proportional to gravity, diffusion still persists in microgravity. Thus, microgravity might act directly or indirectly on a biological system [33,48]. Indirect effects of microgravity on the cellular surrounding have to be considered, such as an optimal distribution of food particles due to the lack of sedimentation. It is important to note that, according to current knowledge, results obtained under hypergravity and 1 g conditions cannot simply be extrapolated to microgravity, as revealed by the existence of thresholds for perception of the stimulus gravity. It is obvious that in many cases the total lack of the stimulus (gravity) is a total new sensation [32].

As a result of the experimental conditions using the 2D clinostat or the aircraft, cells are subjected to irregular stress by cell preparation and handling and - in the case of parabolic flight - by the flight experiment itself. Thus, it is important that the experimental conditions, despite an internal 1 g control always being present, do not alter the cellular and molecular "baseline" beyond an absolute minimum. Therefore, the response to exogenous activators such as PMA (protein kinase C activation) and CD3/CD28 (T cell receptor activation) in 1 g control conditions of the experiment should be nearly the same as during "regular" laboratory conditions. In our study, we detected alterations in the phosphorylation of cdc2, cdc25C and cyclinB1 in response to PMA and CD3/CD28 treatment (Figure 3) and a upregulation of the cell cycle control protein p21 ^Waf1/Cip1 ^(Figure 1). Also during the parabolic flight experiments, cells were responsive to the activator during the 1 g control phase (Figure 5c). Therefore, we conclude that the performed clinostat and parabolic flight experiments fulfil the condition of sufficient cell responsiveness to exogenous stimuli.

The clinorotation experiments analyzing the p21 ^Waf1/Cip1 ^protein expression level clearly shows opposed effects for μg and 1 g (Figure 1). While in control and PMA stimulated cells the amount of protein is increasing over time (1 - 15 min) in simulated weightlessness, it is clearly decreasing in 1 g conditions with progressing time (1 - 15 min). The absence of a comparable result in the clinorotation p21 ^Waf1/Cip1 ^mRNA expression experiment (Figure 2) implies that in even short time clinostat analyses of 5 to 15 min, a translational rather than a transcriptional effect is prevailing. However, experiments performed in real microgravity during parabolic flights show that also effects on p21 ^Waf1/Cip1 ^mRNA transcription are detectable (Figure 5). After 20 s of μg, gene expression levels were increased about 2-fold in control reactions whereas the application of PMA decreased p21 ^Waf1/Cip1 ^expression levels to 1 - 1.6-fold. A time series up to several minutes to compare the development of gene expression with the clinostat results was impossible because the μg time during parabolic flights is restricted to 20 s per experiment.

2D clinorotation is a well established method to simulate weightlessness on Earth [9,21,32,46-52] and its validity has been demonstrated through comparison with data from space experiments, e.g. in the case of T lymphocytes [9,46-51]. However, in our case it is difficult to compare time-points from the different research platforms directly due to technical restrictions. Within the clinostat, activator solutions (such as CD3/CD28 or PMA) have to be added directly before starting clinorotation (in contrast to the technical equipment in the flight hardware allowing the addition of activator solutions directly during the onset of microgravity). Therefore, the 0 min time points represent different conditions: in the clinostat experiments (Figure 3), onset of different gravity conditions (clinorotation vs. 1 g control) and not the time point of addition of PMA or CD3/CD28 as in the flight experiment. Thus, PMA- and CD3/CD28-induced signalling was already active at the 0 min time point in the clinostat and the measured values at 0 min differs between the non-treated and the treated groups. Due to this technical limitation, reliable incubation times using the DLR-clinostat are within a time frame of minutes and therefore much longer than the 22s microgravity provided by the Airbus A300. Thus, it is not possible to validate both research platforms reciprocally, however - as 2D clinorotation is an established simulation method [9,21,32,46-52] - the data from both platforms can be discussed in conjunction, but with caution and an awareness of their limitations. The 20s of the parabolic flight experiments can not be compared directly and kinetically with the 1-10 min time point of the 2D clinostat experiments. For this reason, we only compared μg versus 1 g within the respective experimental platform in order to obtain general information about the gravi-sensitivity of certain molecular reactions. However, the observed gravity-dependent p21 ^Waf1/Cip1 ^regulation could be demonstrated in both platforms.

Several investigations provide evidence of alterations in signal transduction. In lymphocytes, microgravity affected the protein kinase C [10,11], but not the patching and capping of conA-binding membrane proteins [12], influenced NF-kB and MAPK-signaling [13] and the expression of the early oncogenes *c-fos, c-myc *and *c-jun *(summarized in [14]). Other studies demonstrated pro- and antiapoptotic effects of altered gravity in human mononuclear cells [15], human ML-1 thyroid-carcinoma cells [16], and astrocytes [17] and influences on *fas, p53, bax *and *bcl-2 *were described [16,18,20]. A reduced expression of IL-2 receptor [21,22] and a decreased capacity for the production of cytokines [23] were prominent effects of microgravity on T lymphocytes. In microgravity, monocytes lost their capability of secreting IL-1 [24] and expressing IL-2-receptor [25] and demonstrated significant changes in gene induction associated with differentiation of monocytes into macrophages [26], a reduction of phagocytosis and oxidative burst- and degranulation-capacity [27] and massive alterations in the cytoskeleton [28], which in turn influences obvious motility [29]. It seems that not all cell types of the immune system are sensitive to reduced gravity: Extensive studies with natural killer cells in simulated weightlessness and in real microgravity on board the ISS revealed that neither cytotoxic effects nor interferon production is altered in microgravity [30].

In our study we found that p21^Waf1/Cip1 ^mRNA expression is enhanced in microgravity compared to 1 g in CD3/CD28-activated Jurkat T cells and in primary CD4^+ ^- T cells, but also in non-stimulated Jurkat T cells. p21^Waf1/Cip1 ^is one of the p53 target molecules, inhibiting the activity of the cyclinB1/Cdc2 complex [53], leading to a cell cycle arrest. The cell cycle arrest can be induced experimentally by stimulating cells with PMA [54]. PMA activates the protein kinase C, which leads to a p21^Waf1/Cip1 ^upregulation [55,56], a decrease of cyclinB1/cdc2 concentration [57] and subsequent inhibition of the cell cycle at the G1/S and G2/M phase. We further discovered that the histone acetyl-transferase (HAT) inhibitor curcumin abrogated the microgravity-induced overexpression of p21^Waf1/Cip1 ^mRNA, whereas the histone deacetylase (HDAC) inhibitor valproic acid enhanced p21^Waf1/Cip1 ^mRNA expression in microgravity. Interestingly, a recent study found epigenetic alterations in cells exposed to simulated weightlessness and also revealed a decreased expression of a histone deacetylase [58]. Other studies in *long-term *simulated weightlessness found intact T cell receptor signalling [49], but alterations in cell cycle [50] control and upregulation of p21^Waf1/Cip1 ^[51,52]. The p21 ^Waf/cip1 ^gene consists of 492 bp coding sequence emerging from a pre-mRNA of maximally 8682 bp, small enough to function as a regulator for cellular responses via differential gene expression. In human fibroblasts p21 ^Waf/cip1 ^expression levels were increased 8-9 fold only after 30 min of microbeam irradiation [59]. Here we show that gene expression levels are altered even within tens of seconds. The observed overexpression of p21^Waf/cip1 ^is conceivable by rapid transcriptional induction considering the high elongation speed of the RNA polymerase II. While measured elongation velocities reached up to 4.3 kb per min [60], theoretically velocities even above 50 kb per minute have been calculated [61] allowing a significant increase in gene expression after 20s only.

The p21 gene is under the transcriptional control of p53, suggesting that p21 might promote p53-dependent cell cycle arrest or apoptosis [62]. We recently found that p53 was phosphorylated rapidly in proliferating human U937 cells during the microgravity phase of parabolic flights [63]. Therefore, a role of p53 in microgravity-dependent p21-expression can be assumed. However, the rapid appearance of p21 RNA during 20 seconds of microgravity suggests a more direct effect of altered gravity on p21 transcriptional regulation.

During human space flight, human cells are exposed to microgravity and the radiation of space. p21^Waf1/Cip1 ^is a master effector of multiple anti-proliferative pathways [64] reacting e.g. on cellular DNA damage induced by radiation [59,65]. Because p21 is obviously essential for cell cycle arrest induction after radiation [62,66] and radiation increased the expression of p21 [66], a key role of p21 in radiation-induced cell cycle arrest in the cellular response to DNA damage [67] has been suggested. On the other side, it has been shown that ionizing radiation induces ubiquitin-dependent degradation of p21Cip1 and therefore contributes to the survival of neoplastic cells after ionizing radiation [67]. We suggest that microgravity-induced transcriptional induction of p21 represents a possible mechanism of synergistic effects between microgravity and space radiation which could finally contribute in cell cycle arrest, enabling DNA repair.

Interestingly, DNA microarray analysis from rat muscle RNA during the NASA STS-90 Neurolab spaceflight mission revealed an inhibition of genes for cell proliferation compared to 1 g ground controls, supporting also an *in vivo *down-regulating effect of space flight on cell cycle progression [68]. Recent studies investigated the effect of systemic stress induced by microgravity or space flight and their mediators on the human immune system [69,70]. Our results suggest that microgravity is perceived by cells of the human immune system as a stress signal which is then translated into rapid intracellular responses. Identifying gravi-sensitive signal transduction pathways in cells of the immune system will help to find appropriate targets for therapeutic intervention or preventive countermeasures related to the immune system of astronauts during long-term space missions. As a result of our study, we suppose that cell cycle progression in human T lymphocytes requires Earth gravity. Additionally, we found that microgravity-induced overexpression of p21^Waf1/Cip1 ^mRNA was abrogated by the histone acetyltransferase inhibitor curcumin, a phenolic compound from turmeric (*Curcuma longa *L.), a member of the ginger family (Zingiberaceae), an approved food additive. Rapid induction of cell cycle arrest signaling in microgravity could represent a protective cellular reaction to enable DNA repair, energy saving and recovery after gravitational stress.

## Abbreviations

AIQ: 5-Aminoisoquinoline; BC: Biomedical Science Support Center; CU: Curcumin; HAT: Histone acetyl-transferase; HDAC: Histone deacetylase; ISS: International Space Station; MACS: Magnetic Activated Cell Separation; μg: Microgravity; PBMC: Peripheral Blood Mononuclear Cell; PBS: Phosphat-buffered saline; PKC: Proteine kinase C; PMA: 12-O-Tetradecanoylphorbol-13-acetate; STS: Space transportation system; TCR: T cell receptor; VP: Valproic acid

## Competing interests

The authors declare that they have no competing interests.

## Authors' contributions

OU developed the study idea, concept and the overall study design as well as planned, coordinated and supervised the study, and wrote and edited the manuscript. CT, GB, CD and KP carried out the experiments and contributed to the manuscript. CT contributed to the study idea and overall study design, performed the qRT-PCR analysis and contributed to the manuscript. GB and KP planned, coordinated and supervised the biological operations during the parabolic flight campaigns. KP also participated in the study design and coordination. KL, ST and NG carried out the clinostat experiments and sample analysis. KP contributed to the analysis of clinostat experiments. AA, UZ, CD, JB, ST, SC and FZ contributed to the parabolic flight experiments. ST participated in the development and improvement of the in-flight-procedures. AH and FE developed and constructed the experimental system for cell culture experiments on board of the Airbus 300 ZERO-G. OU, KP and ST contributed to this development. AH and FE coordinated, performed and supervised the technical operations during the parabolic flight campaigns. KHG contributed to the technical development and to the manuscript. AC contributed significantly to the study idea. FZ, RH and AC participated in the development of the study idea and design and contributed to the manuscript. RH participated also in the ground-support of parabolic flight experiments and provided the ground-facilities (clinostat). All authors read and approved the final manuscript.

## Supplementary Material

Additional file 1 **Short technical description of in-flight-hardware**. A short summary of the most important technical parameters of the in-flight-hardware such as rack configuration, weight, dimensions and power supply. Click here for file
