# Supplementary material for: Rapid alterations of cell cycle control proteins in human T lymphocytes in microgravity
Source: Cell Commun Signal. 2012 Jan 24;10:1. doi: 10.1186/1478-811X-10-1 (PMC3275513; doi:10.1186/1478-811X-10-1)
Supplement: Additional file 1 — Short technical description of in-flight-hardware. A short summary of the most important technical parameters of the in-flight-hardware such as rack configuration, weight, dimensions and power supply. [file 1478-811X-10-1-S1.DOC]

**Additional file 1: Short technical description of in-flight-hardware**

In take-off-configuration, the *storage rack* has a mass M=145kg, 4 attachment points to the aircraft cabin rail (each attachment point carries a mass of 36.25kg) with a distance between two attachment points of 990mm. The load over one linear meter supported by the aircraft rail is 36.25+36.25=72.5kg<100kg (maximum allowed linear load). This rack has a capacity of 60 cell containers and keeps a constant temperature of 37 °C by five silicon heating plates connected to an automatic control loop. The basic structure of the store module consists of an aluminium casting profile and the side panels disguised by a sandwich construction with isolation material between the outside plastic plate and an internal welded aluminium box. A bimetal safety switch protects from overheating. The *working rack* has a mass M=240kg, 4 attachment points to the aircraft cabin rail (each attachment point carries a mass of 60kg) with a distance between two attachment points of 1118mm. The load over one linear meter supported by the aircraft rail is 60kg<100kg (maximum allowed linear load). In the range A (4 °C) are three separate hose pumps, which pump one of the two possible stop liquids (e.g. ethanol or 4% formaldehyde) into the cell containers. The pumps are activated and regulated by the control unit inside range C, which carries also all electrical connections and fuse elements. All liquids are sucked under exclusion of air. Inside range B (37 °C) are three bags with activator liquids, connected to three separate hose pumps, which pumps the activator solution into the cell container upon activation of the individual experiment. Constant temperature of 37 °C is kept by five silicon heating plates connected to an automatic control loop. The housing of range B is a welded aluminium box. Range D is the actual working space with three cell container in parallel, which are connected to the activator and fixation liquid bag. Range D is waterproof and the door position is controlled by a safety switch (sensor). Activation of the experiment is only possible when the front door is closed. All liquids inside are totally enclosed in the hose bag and the waterproof rack walls (welded aluminium box) and doors. The frame construction of the cooling range exists of aluminium casting profiles, outside plastic walls, a welded and waterproof aluminium box and glued isolation material. The dimensions of the rack are 600x1200x1461mm. The *cooling rack* has a mass of M=110kg, four attachment points to the aircraft cabin rail (each attachment point carries a mass of 27.5kg) with a distance between two attachment points of 508mm. The load over one linear meter supported by the aircraft rail is 55kg<100kg (maximum allowed linear load). The system is constructed to withstand a structural load of 9g in the event of an aircraft crash. The cooling rack consists of aluminium casting profiles, outside plastic walls, inside welded aluminium box and isolation material. Cooling is performed by two Peltier cooling elements (24 VDC plate cooler without use of any cooling fluidities). The heat resulting from the cooling process is transferred to the aircraft cabin environment by fans. Temperature of 4 °C is kept over an automatic control loop. The dimensions of the rack are 717x1192x681mm. All racks have a total weight of 495 kg and a maximal in-flight-power consumption of 1750 W. All 60 individual cell containers possess a double wall, which is realized by an internal sterile biocompatible bag (Nutrimix, Braun, Melsungen, Germany) carrying the cell suspension and culture medium, totally enclosed in an air- and liquid-proof plastic container. A self-closing air valve at the rear of each container allows the air to escape during the filling process. Before and after each flight, the entire tube system of the working rack is rinsed by sterile water, ethanol and sterile PBS. All pumps were tested and calibrated directly prior each flight.
